# Leaf economics of deciduous and evergreen plants: how do they exhibit trait optimization under resource variations and environmental constraints

**DOI:** 10.1007/s00442-025-05734-z

**Published:** 2025-06-05

**Authors:** Kouki Hikosaka, Yusuke Onoda, Kaoru Kitajima

**Affiliations:** 1 Graduate School of Life Sciences, Tohoku University, Aoba, Sendai 980-8578 Japan; 2 Graduate School of Agriculture, Kyoto University, Kyoto, 606-8502 Japan

**Keywords:** Leaf economics spectrum, Leaf habit, Leaf lifespan, Optimization theory, Return on investment

## Abstract

Leaf habit, whether a plant is deciduous or evergreen, is one of the most conspicuous traits in plant species, ecosystems, and biomes. In this review, we discuss theories and empirical patterns of deciduous and evergreen habits with a focus on the optimal strategies. Historically, optimal leaf habits and leaf lifespans (LL) have been explained from the viewpoints of carbon economy and nutrient use. In the leaf economics spectrum, deciduous and evergreen species are positioned at the resource-acquisitive and resource-conservative sides of the spectrum, respectively. Seasonal variations in leaf traits of deciduous species could be understood simply as optimization of carbon economy in relation to the progress of the growth season. Evergreen species, however, exhibit more complex patterns, which could be understood as acclimation to seasonal environmental changes or resource allocation to leaf replacement and reproduction. Optimal timing of leaf shedding in evergreen species varies depending on the limiting factor for leaf retention. The return on investment (RI) through the lifetime of a leaf should be greater in evergreen species when there is no environmental suppression. However, RI of evergreen species is often suppressed by seasonal environment or species interaction. Deciduous species can have comparable RIs with evergreen species when they can occupy better environment such as gaps in forests. In conclusion, which leaf habit is advantageous can be understood from trait optimization perspectives. Such knowledge should help forecast how forest ecosystems will respond to changing climate and other anthropogenic environmental impacts.

## Introduction

**Fig. 1:**
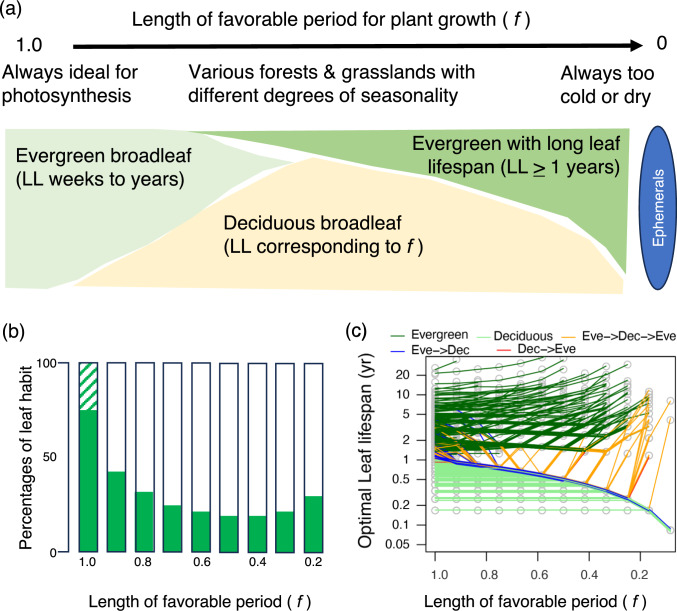
The relative dominance of evergreen and deciduous plants relative to the favorable duration for photosynthesis in a year (f). **a** A conceptual diagram. The value of *f* = 1.0 is found in equatorial tropics (lowland to lower montane) dominated by evergreen broadleaf trees, which may have a leaf longevity of just a few months (e.g., many pioneer trees and herbaceous plants) to multiple years (late successional trees and their juveniles in shade). The value of *f* = 0 is found in Antarctica or very dry deserts, which may occasionally harbor ephemeral plants. When *f* has intermediate values, deciduous species may coexist with evergreen species. Evergreen species at short *f* values have leaves that live for multiple years to pay back the leaf construction cost. Altitudes and local edaphic conditions may modulate the *f* values at a given latitude. **b** Simulation by Kikuzawa (1991) on the relationship between the *f* values and the percentage of leaf habit of tree species predicted by his model (hatched: evergreen with very short lifespans, closed: evergreen with leaf lifespan (LL) > 1 year, open: deciduous). **c** The simulated optimal LL relative to various *f* values by Kikuzawa et al. (2013). Each line represents an arbitrary trait combination derived from the Glopnet database. With a decrease in *f*, the deciduous trait combinations (LL < 1) tend to decrease LL, whereas evergreen trait combinations (LL ≥ 1) tend to increase LL. The simulation also predicts that some species that have greater than a 1-year LL at *f* = 1 become deciduous at an intermediate *f*, then return to evergreen at a short *f* (Eve → Dec → Eve). Also, some species changes from evergreen to deciduous (Eve → Dec) or from deciduous to evergreen (Dec → Eve). **b** was redrawn from Kikuzawa (1991) with modifications

Leaf habit, whether a plant is deciduous or evergreen, has been recognized throughout the history of plant ecology and phytogeography, including studies of the latitudinal gradient of the leaf habit (von Humboldt and Bonpland 1807; Schimper 1903; Whittaker 1962; Kikuzawa and Lechowicz 2011). For a long time, it has been widely accepted that a leafless season corresponds to a very cold or dry season that poses a risk of freezing or drought damage. Scientists are eager to provide a mechanistic explanation for observed patterns of leaf habits (e.g., Givnish 2002), which are often linked to leaf lifespan (LL), as deciduous trees typically have a shorter LL compared with evergreen trees. A notable exception is found in seasonal humid tropics where some evergreen pioneer species have a short LL of one to several months (Kikuzawa 1991) (Fig. 1). The evergreen habit may be favored on infertile soils within climate zones that are otherwise dominated by deciduous trees (Monk 1966). Thus, LL varies widely among species within a given climate (Russo and Kitajima 2016). A seminal review by Chabot and Hicks (1982) entitled “Ecology of Leaf Life Spans” lists a multitude of hypotheses that attempt to explain species differences in leaf habits and LL.

Do species differences in leaf habits represent fundamental differences in physiological strategies between deciduous vs. evergreen plants? Here, we consider this classic question and review theories and empirical trait patterns of deciduous and evergreen species in a comprehensive manner. Firstly, we revisited earlier studies that addressed the differences among species in LL and other leaf traits from economic viewpoints to understand the ecophysiological differences between the two leaf habits. Then, we discuss carbon economy models related to optimal LL and nutrient use efficiency, both of which are important for understanding the significance of leaf habits. We further discuss the adaptation of leaf function in changing environments based on theories of nitrogen use in leaves. We also review how observed LL and leaf habits might reflect phenotypic plasticity vs. interspecific differences, including triggers that induce leaf shedding. Finally, we discuss the coexistence and replacement of species with different leaf traits from the viewpoint of return on investment (RI).

## Development of economic viewpoints for understanding ecological differences between leaf habits

Beginning in the 1970s, many researchers attempted to address the physiological reasons that explain leaf habits in seasonally dry environments (Mooney and Dunn 1970; Orians and Solbrig 1977; Lerdau et al. 2023). Progress in plant population ecology and evolutionary biology around this time also prompted the development of cost–benefit perspectives at leaf and whole-plant levels (e.g., leaf carbon gain model by Chabot and Hicks 1982). A common assumption in the cost–benefit approach is that plants achieving greater carbon gain or nutrient use efficiency outcompete their neighbors. Another assumption is that the modular nature of plants suggests that better photosynthetic performance of individual leaves results in better growth of the whole plant and ultimately a higher population growth rate. During the 1970s and 1980s, a need to distinguish proximate vs. ultimate causes in explaining a particular phenological pattern became evident. It was widely accepted that a deciduous habit reflects the evolutionary advantage of avoiding seasonal risks from freezing damage or drought-induced embolism. In autumn, the leaves of winter-deciduous species resorb nutrients before the risk of freezing becomes eminent using day length or diel fluctuation of temperature as a signal. In seasonally dry tropics, dry-deciduous trees sense water stress and drop leaves before plants suffer severe embolism (Reich and Borchert 1984). In the spectrum of avoidance-to-tolerance during the cold winter or prolonged dry season, the evergreen habit can conserve nutrients without frequent loss of leaves and may be advantageous, particularly when there is limited nutrient availability.

**Fig. 2:**
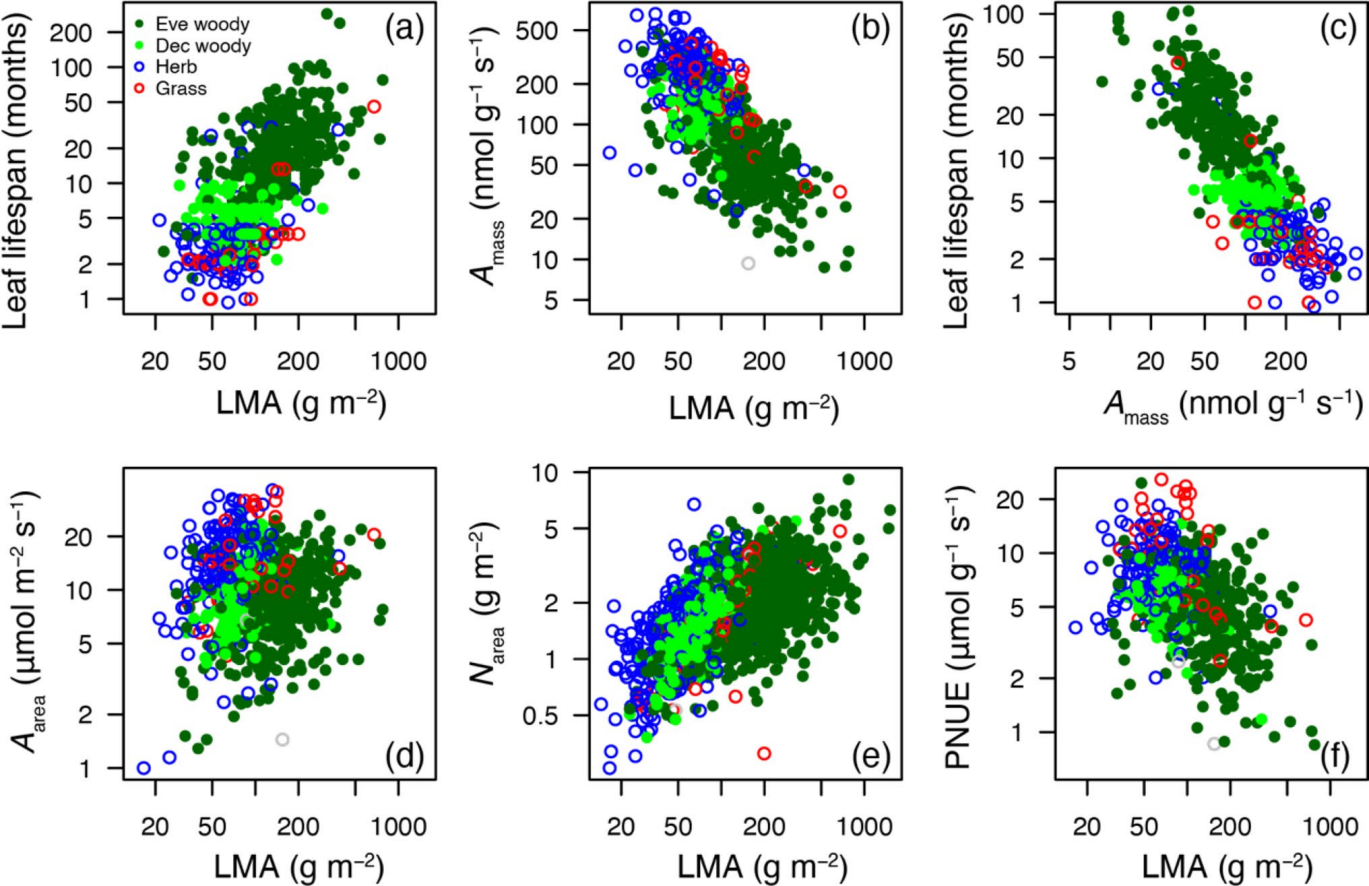
The leaf economics spectrum and leaf trait correlation patterns based on 2,548 species from 175 sites (Wright et al. 2004). Leaf mass per area (LMA) is positively correlated with leaf lifespan (LL) (**a**) and negatively correlated with mass-based photosynthetic rates (*A*_mass_) (**b**). Thus, a tight negative correlation is observed between LL and *A*_mass_ (**c**). Area-based photosynthetic rates (*A*_area_) are not correlated with LMA (**d**), whereas *N*_area_ is positively correlated with LMA (**e**). The ratio of *A*_area_ to *N*_area_, known as the photosynthetic nitrogen use efficiency (PNUE), is negatively correlated with LMA (**f**)

By the mid-1980s, plant ecophysiology became a mature field with the accumulation of data on photosynthetic characteristics and related leaf traits from various environments and life history types. The “Physiological Ecology” monograph series (Academic Press) edited by Mooney, Bazzaz, Chapin, Ehleringer, Pearcy, and Caldwell (all of whom served on the editorial board of Oecologia) has been useful in establishing the foundational knowledge in the field. Pooling leaf trait data from annuals to evergreens, Field and Mooney (1986) found a strong correlation between mass-based photosynthesis (*A*_mass_) and leaf nitrogen concentration (*N*_mass_). They also demonstrated that the *N*_mass_ is lower in leaves with a higher leaf mass per area (LMA). Peter Reich and colleagues, who viewed LL as key to plant strategies, analyzed leaf traits in various biomes (Reich et al. 1991a, 1992, 1997). They observed tight correlations among multiple leaf traits within and across biomes, which resulted in a global database of leaf traits for more than 2000 species (Glopnet) led by Ian Wright. These efforts introduced the notion of a worldwide “leaf economic spectrum (LES)” (Wright et al. 2004). This spectrum runs from fast- to slow return on the investment of nutrients and dry mass in leaves, corresponding to the gradient of short to long LL (Fig. 2). The correlation exists irrespective of growth forms, plant functional type, and biomes. Deciduous species are positioned on the relatively fast return side compared with the evergreen species (Wright et al. 2005a). There is a substantial overlap between deciduous and evergreen habits along the LES, because there are evergreen species with short LL of less than 1 year, and brevideciduous species that lose leaves during a short period of a few weeks to a month when they exchange all of their leaves. Thus, when we see variations in leaf functional traits, the deciduous habit is not essentially different and can be included in the evergreen habit as one of the extreme variations in leaf longevity.

## Carbon economy models and optimal leaf lifespan

Mooney and coworkers (Mooney and Dunn 1970; Millar and Mooney 1974) demonstrated that drought-deciduous species have shorter LL, but higher photosynthetic rates compared with evergreen species, such that annual carbon gains are similar between deciduous and evergreen species under Mediterranean climate. The idea of a trade-off between daily photosynthetic income and LL is at the core of the cost–benefit model described by Chabot and Hicks (1982), which assumes that a leaf as an organ for photosynthesis must optimize net carbon gain as described by the following equation:

1 $${\text{Net carbon gain}} = \sum P_{fi} F - \sum P_{ui} U - C - W - H - S$$

where *P*_*fi*_ and *P*_*ui*_ are the daily photosynthetic incomes of the leaf at age *i* (days) during the favorable and unfavorable seasons, respectively, and *F* and *U* are the duration of the favorable and unfavorable seasons, respectively, *C* is the initial cost of producing the leaf (gram equivalent of photosynthates), *W* is the loss due to seasonal stress, *H* is the loss to herbivores, and *S* is the photosynthate stored in the leaf. This model may be used for the whole leaf, unit leaf area, or unit leaf mass. Regardless of the normalization, this model suggests that when the construction cost per unit leaf area is high (i.e., high LMA), the leaf should have a much higher photosynthetic capacity or longer LL. Unfortunately, this model has too many variables to parameterize. Furthermore, the model of Chabot and Hicks (1982) does not explain the bimodal pattern of evergreen dominance along the latitude (DeFries et al. 2000).

**Fig. 3:**
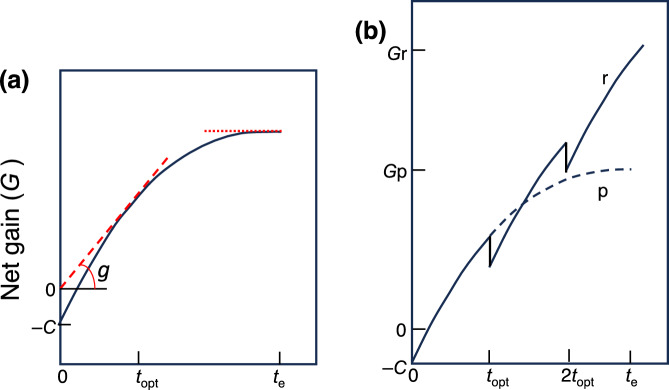
Time dependence of carbon gain and the optimal leaf lifespan (LL) in the Kikuzawa model (1991). Net carbon gain at time 0 is negative because of the construction cost (− *C*) and increases rapidly at first and then gradually because of a decrease in photosynthetic rate with time. If there is no constraint, the optimal LL is *t*_e_, at which the photosynthetic rate is zero (**a**). If there is a limitation in the leaf number for an individual, the optimal LL is *T*_opt_ because replacement by a new leaf results in the higher carbon gain for the individual (*G*r) than compared with retaining an old leaf (*G*p) (**b**). Redrawn from Kikuzawa (1991) with modifications

Kikuzawa (1991) simplified this model by assuming gross photosynthetic rates during the unfavorable time to be zero and ignoring any loss due to stress, herbivory, and storage, except for the maintenance respiration rates. The equation is as follows:

2 $$G = g \times t = \mathop \smallint \limits_{0}^{t} p\left( t \right) dt - \mathop \smallint \limits_{0}^{t} m\left( t \right)dt - C$$

where *G* is the total carbon gain during the LL, *g* is the marginal gain, *p*(*t*) is the daily photosynthetic rate at leaf age *t*, *m*(*t*) is the maintenance cost of the leaf at age *t*, and *C* is the initial construction cost (Fig. 3a). The duration of the favorable time as a fraction of a year (*f*) may be introduced in this equation. He further introduced the following two important assumptions. One is the age-dependent decrease of the photosynthetic rates and respiration rates, which enables numerical simulations of the model. The other one is the limitation of the number of leaves per plant. Leaf number increases with time when the plant is small and growing without resource constraints, but it cannot increase infinitely because of the mutual shading of the leaves, biomechanical load, and/or nutrient deficiency. If there is no such limitation and the plant can retain infinite number of leaves, leaves should be retained until their net carbon gain (*G*) becomes maximum (dotted line in the Fig. 3a). In contrast, if the number of leaves is limited, an old leaf should be replaced by a new leaf when *g,* rather than *G,* is maximized (slope of the broken line in Fig. 3a is maximum), which increases the whole plant photosynthesis (*G*r relative to *G*p in Fig. 3b). If unfavorable period is ignored, the optimal LL (LL_opt_) that maximizes *g* is calculated as follows:

3 $${\text{LL}}_{\text{opt}}=\sqrt{\frac{2bC}{a-m}}$$

where *a*, *b,* and *m* are the initial photosynthetic rate, the day when the photosynthetic rate becomes zero, and the maintenance respiration rate, respectively. The outputs of Kikuzawa model can explain the bimodal pattern of evergreen dominance along the latitude (Fig. 1). In aseasonal environments (large value of *f*), evergreen species with various LLs can dominate. With a shortening in favorable period, the proportion of evergreen trees decreases to reach a minimum at intermediate values of *f*, then it can increase again with a further decrease in the length of the favorable period, which can explain the bimodal pattern of evergreen dominance along the latitude.

Two decades after this model was published, Kikuzawa et al. (2013) conducted a numerical simulation for optimal LL maximizing *g* for various lengths of the favorable periods using actual leaf trait values from the Glopnet database. The results showed how the relative dominance of deciduous and evergreen species changes with the latitudinal variations in growing season length (Fig. 1). They also showed that the Kikuzawa model can reproduce the patterns observed by Wright et al. (2005b), in which deciduous species have a shorter LL with decreasing mean annual temperature (MAT), whereas evergreen species have a longer LL with decreasing MAT. The latter is predicted because it requires a longer time to pay back the construction cost and to achieve the optimal carbon gain. In addition, they also show that the correlation patterns of the key leaf traits, namely the LMA, photosynthetic rate, and respiration rate, can be shaped by the model of leaf carbon economy (Kikuzawa et al. 2013). More recently, incorporating environmental responses of leaf physiology into the Kikuzawa model, Wang et al. (2023) showed that the latitudinal trend of not only the optimal LL, but also the optimal LMA differs between the evergreen and deciduous plants, the latter of which is consistent with the community-weighted mean of LMA in actual plant communities.

## Nutrient use efficiency

Nutrient use strategy is also relevant to the underlying mechanism for the niche differentiation between deciduous and evergreen plants. In particular, nitrogen use has been focused because it tends to limit plant growth in many ecosystems (Aerts and Chapin 2000). The relations of leaf habits with soil nutrient availability were reported by Monk (1966), who quantified the abundance of evergreen and deciduous species in Florida forests with different soil fertility. He found that evergreen species are more abundant in dry and infertile soils and postulated that the evergreen habit reduces the inevitable nutrient loss that accompanies leaf loss; thus, it is an advantage in infertile habitats. Similar conclusion was reached from the perspective of nutrient use efficiency by Small (1972), who built upon the concept of nutrient use efficiency for biomass production. He calculated nitrogen use efficiency (NUE) as the net photosynthetic rate per unit leaf nitrogen (now known as photosynthetic nitrogen use efficiency, PNUE) × LL × 1/(1-*R*), where *R* is the fraction of resorbed nitrogen (resorption efficiency). LL × 1/(1-*R*) corresponds to the mean residence time of nitrogen in the plant body (see below). He showed that NUE is much higher in evergreen compared than in deciduous species and argued that the evergreen habit is advantageous in infertile habitats because of a longer duration to amortize the absorbed nutrients (Small 1972); however, this result was criticized because such a trend was not observed in other ecosystems (Chapin et al. 1980).

Chapin (1980) defined NUE as biomass production per unit nitrogen content at the plant level and suggested that the inverse of plant nitrogen concentration may be a proxy for NUE. From a literature review, he concluded that when grown under the same condition, plants from nutrient-poor habitats exhibited higher nitrogen concentrations. This pattern means that they are less efficient for nitrogen use compared with plants from nutrient-rich habitats, but his NUE calculation ignored nitrogen resorption from senescing leaves (Killingbeck 1996). Vitousek (1982) redefined NUE as biomass production per unit of nitrogen taken up (as done by Hirose 1975) and suggested that the inverse of nitrogen concentration of litterfall can be used as a proxy for NUE. In a mature forest stand, in which standing biomass and nutrient contents are stable, biomass production and nitrogen uptake may be considered equal to the loss of biomass and nutrients from the plant body, respectively. Vitousek (1982) collected data on the nitrogen concentration of litterfalls and found that NUE is higher in nutrient-poor habitats.

Berendse and Aerts (1987) showed that NUE consists of two components, mean residence time of nitrogen in the plant body (MRT) and instantaneous rate of biomass production per unit of nitrogen in the plant body. With respect to the latter, the two correlations have been widely observed: (1) leaf photosynthetic rate is strongly correlated with leaf nitrogen content (Mooney et al. 1981; Gulmon and Chu 1981) and (2) plant growth rate is strongly correlated with plant nitrogen concentration (Ingestad 1979). Growth rate (biomass production rate) per unit nitrogen content is also known as nitrogen productivity (NP; Ingestad 1979). NUE may be expressed as follows:

4 $$\text{NUE}=\frac{\Delta M}{\Delta N}=\frac{\Delta M}{N\Delta t}\times \frac{N\Delta t}{\Delta N}$$

where Δ*M* is the biomass production during Δ*t*, Δ*N* is the nitrogen uptake (or loss) during Δ*t*, and *N* is the amount of nitrogen in the plant body. The first term represents NP, and the second term represents MRT. MRT reflects LL and nitrogen resorption efficiency (*R*). The latter is defined as the fraction of nitrogen resorbed from the living leaf before death and is given by LL × 1/(1 − *R*), as mentioned above. Although there is a large variation in the nitrogen resorption efficiency among species and growth conditions, there are no significant differences between deciduous and evergreen species (Aerts and Chapin 2000; Vergutz et al. 2012). Conversely, experimental studies indicated that MRT is positively correlated with LL (Aerts 1990; Escudero et al. 1992; Eckstein and Karlsson 1997; Aerts and Chapin 2000; Hikosaka et al. 2021).

Deciduous and evergreen species in a heathland were shown to have a similar NUE; however, NP was higher in deciduous species, whereas MRT was longer in evergreen species (Berendse and Aerts 1987; Aerts 1990). These differences were confirmed by Eckstein and Karlsson (1997), who showed that 14 species coexisting in a subarctic environment had a similar nutrient use efficiency (both for nitrogen and phosphorus), but there was a trade-off between NP and MRT across the species. A simulation of plant productivity with the same NUE but under different NP and MRT by Aerts and van der Peijl (1993), showed that plants with higher MRT exhibited higher biomass production when nitrogen availability limited plant growth over time. Therefore, natural selection in nutrient-poor habitats does not necessarily favor a high NUE strategy, but rather a strategy that reduces nutrient losses, whereas natural selection in nutrient-rich habitats favors plants with high biomass production rates (Berendse and Aerts 1987; Aerts and Chapin 2000).

## Optimization of nitrogen allocation to maximize biomass production

**Fig. 4:**
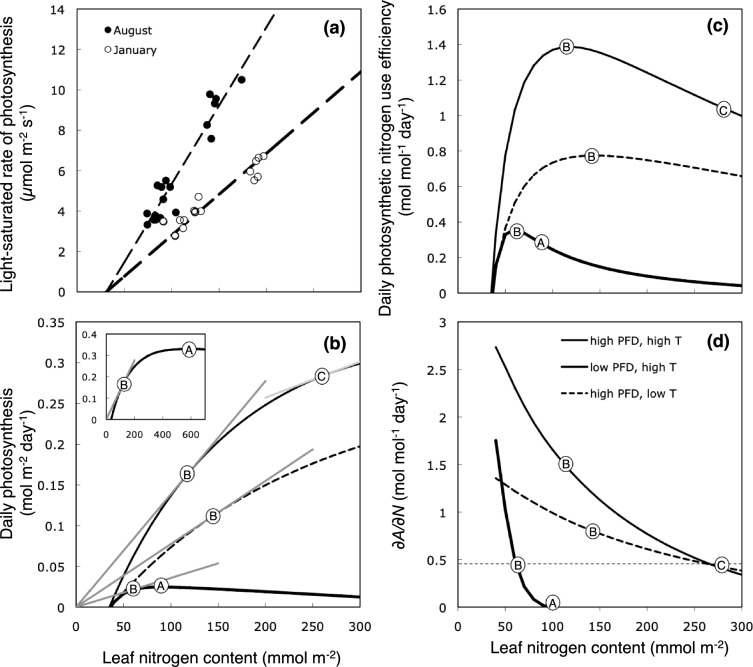
Dependence on leaf nitrogen content for the light-saturated rate of photosynthesis (**a**), daily carbon exchange (**b**), daily photosynthetic nitrogen use efficiency (PNUE), (**c**) and slope of the tangent for the daily carbon gain (∂*A/*∂*N*, **d**) in *Aucuba japonica*. Closed and open symbols in **a** are data obtained in the summer (August) and winter (January) at the growth temperature. Daily carbon gain, PNUE, and ∂*A/*∂*N* were calculated from the data shown in **a**. Thin continuous, broken, and thick lines in **b**, **c,** and **d** represent the values in summer under high-light conditions (HT-HL), those during winter high-light conditions (LT-HL), and those at summer under low-light conditions (HT-LL), respectively. The circle A and B denote the leaf nitrogen content that maximizes daily carbon gain and daily PNUE, respectively. In circle C, the ∂*A/*∂*N* of the HT-HL leaves is identical to that of the HT-LL leaves, indicating optimal allocation of nitrogen between these two leaves. Redrawn from Hikosaka et al. (2014) with modifications

Earlier studies demonstrated a strong correlation between the light-saturated rate of photosynthesis and leaf nitrogen content (Mooney et al. 1981; Gulmon and Chu 1981; Fig. 4a). This is because more than half of the leaf nitrogen may be invested in the photosynthetic apparatus, particularly ribulose-1,5-bisphosphate carboxylase/oxygenase (rubisco), a key photosynthetic enzyme (Evans 1989; Makino and Osmond 1991; Hikosaka and Terashima 1996). Mooney and Gulmon (1979) considered nitrogen and photosynthesis as the cost and benefit of carbon gain, respectively. This resulted in the concept that plants should allocate nitrogen efficiently to maximize the marginal carbon gain (slope of the tangent line of the photosynthesis-nitrogen curve).

While the light-saturated photosynthetic rates typically show a linear correlation with leaf nitrogen content, the daily photosynthetic rates or light-limited photosynthetic rates typically show a convex curve (Fig. 4b). The shape of the curve is strongly influenced by irradiance, such that photosynthetic rates at a given nitrogen content is lower at lower irradiance (Fig. 4b), indicating that the marginal carbon gain is smaller at lower irradiance. Field (1983) found that the PNUE of the whole canopy (or individual) is maximized when nitrogen is allocated so that the tangent *λ* of the daily photosynthesis-nitrogen curve is identical across the leaves based on the following equation:

5 $$\lambda =\frac{\partial A}{\partial N}$$

where *λ* is the Lagrange multiplier and *A* is the daily photosynthesis. This equation means that more nitrogen should be allocated to leaves exposed to higher irradiance (Fig. 4d). Hirose and Werger (1987b) combined Field’s theory with canopy photosynthesis and demonstrated the importance of nitrogen allocation to maximize canopy photosynthesis. This optimization theory was further developed in various studies (Farquhar 1989; Anten et al. 1995; Hikosaka 2014); however, it should be noted that actual nitrogen distribution is close, but not identical, to the optimum. Hikosaka et al. (2016a) showed that in most canopies, the slope of actual nitrogen distribution is half of the optimum. Although several studies proposed hypotheses as to why nitrogen distribution is suboptimal, there was no obvious explanation (see Hikosaka 2016 for review).

The optimal timing of leaf shedding may also be explained based on nitrogen use. If carbon economy alone is considered, we can expect that the leaf should be shed when the carbon gain becomes negative because of aging or over-shaded by other leaves as predicted by the Chabot and Hicks model (the optimal timing that maximizes *G* rather than *g*; see above). However, if nitrogen use is more important, plants may drop old leaves that make positive carbon gain if it improves whole-plant photosynthesis (Franklin and Agren 2002). Hikosaka (2003) combined the theories of canopy photosynthesis and nitrogen use and proposed a simulation model, which well explained environmental responses of leaf dynamics (Hikosaka 2005). Escudero and Mediavilla (2003) presented a simple model in which the whole-plant carbon gain is maximized by shedding older leaves only when photosynthetic income gained by re-translocated nitrogen in new leaves exceeds the photosynthesis of the leaves lost. They expressed this idea by the following simple inequality:

6 $${\text{PNUE}}_{{{\text{old}}}} /{\text{PNUE}}_{{{\text{new}}}} < R$$

where PNUE_old_ and PNUE_new_ represent the daily (not instantaneous) NUE of photosynthesis (daily carbon gain per unit of leaf nitrogen) of the old and new leaves, respectively, and *R* is the nitrogen resorption efficiency. The old leaf should be shed when the above inequality is satisfied. The application of this model is discussed later in this review.

Optimization of leaf nitrogen content may also be considered at the leaf level. There are two ‘optimal’ nitrogen contents (Hikosaka et al. 2014; Hikosaka 2016). One maximizes daily photosynthesis per unit leaf area (**A** in Fig. 4b–d) and the other maximizes daily photosynthetic photosynthesis per unit leaf nitrogen (daily PNUE) (**B** in Fig. 4b–d) (Hirose 1984; Hirose and Werger 1987a; Hikosaka and Terashima 1995). When plant growth is limited by nitrogen uptake, it is maximized if the leaf nitrogen content follows the case **B** (Hirose 1988; Hilbert 1990; Ishizaki et al. 2003). Hikosaka and Terashima (1995) reported that nitrogen content in actual plants is closer to cases **A** and **B** when nitrogen availability is high and low, respectively. The optimal nitrogen content that maximizes daily PNUE (**B**) is correlated with the real leaf nitrogen content in field plants in a changing environment (Hirose and Werger 1987a; Anten et al. 1998; Muller et al. 2011); however, the optimal nitrogen content is not necessarily the same as the nitrogen content that maximizes whole-plant photosynthesis (pattern **C** in Fig. 4b–d). Because natural selection optimizes whole-plant performance, it is unclear why leaf nitrogen content appears to be optimized for leaf-level photosynthesis rather than whole-plant photosynthesis. This is a long-standing enigma.

## Trait variations due to phenotypic plasticity vs. species differences

**Fig. 5:**
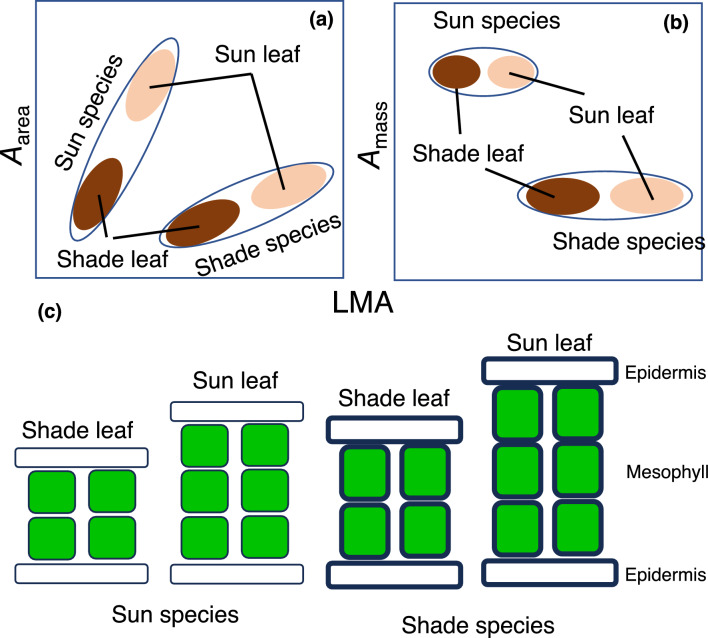
Diagrams for within- and between-species variation leaves in response to a light environment. The relationship between photosynthetic rate per unit area (*A*_area_) and LMA (**a**) and the relationship between photosynthetic rate per unit mass (*A*_mass_) (**b**) in sun- and shade-acclimated leaves of the sun (shade-intolerant) and shade (shade-tolerant) species. Vertical section of leaves (**c**). Within a species, the sun leaves have a thicker mesophyll compared with the shade leaves. Shade species have a greater leaf thickness and cell density compared with the sun species

One of the most widely studied topics in leaf photosynthesis is acclimation to a light environment, in which acclimation to greater light results in a higher photosynthetic rate and nitrogen content on a leaf area basis (*A*_area_ and *N*_area_), greater LMA, and shorter LL (Boardman 1977; Björkman 1981; Gulmon and Chu 1981; Fig. 5a, b). The mass-based photosynthetic rate and nitrogen concentration (*A*_mass_ and *N*_mass_) and PNUE are largely similar (Gulmon and Chu 1981; Fig. 5) between sun and shade leaves. These trends are not necessarily consistent with LES, in which species with a higher *A*_mass_ have a higher *N*_mass_, smaller LMA, and shorter LL, with whereas much weaker correlations exist between area-based traits (Wright et al. 2004, 2005a; Onoda et al. 2017; Fig. 2). In tropical areas, sun-adapted (e.g., light-demanding, heliotropic, or shade-intolerant) species tend to have a higher *A*_mass_ compared with shade-adapted species (Kitajima 1994; Fig. 5a, b), indicating that trait variations are different between light acclimation and adaptation.

Within-species changes (i.e., acclimation or phenotypic plasticity) in LMA are largely driven by leaf thickness and to a minor degree by leaf density (Kitajima et al. 2013; Fig. 5c). Acclimatory changes in leaf thickness are associated with photosynthesis rather than biomechanics. *A*_area_ is generally limited by the reactions of the Calvin cycle enzymes or thylakoid electron transport (Farquhar et al. 1980; von Caemmerer and Farquhar 1981). Therefore, plants need to invest more nitrogen to increase photosynthetic machinery, particularly in rubisco per unit area, which underpins the correlation between *A*_area_ and *N*_area_ within species. With a high *A*_area_ and *N*_area_, CO_2_ diffusion and leaf anatomy become limiting factors. Because CO_2_ diffusion is very low in the liquid phase, chloroplasts must be located near the cell membrane and are flatter to minimize the CO_2_ pathlength from the mesophyll cell surface to the carboxylation site (Evans et al. 1994; Terashima et al. 2001). Increasing leaf thickness creates the space necessary for increasing the mesophyll surface per unit leaf area (known as A^mes^/A; Nobel et al. 1975). Without such intracellular localization and morphological acclimation, chloroplasts cannot receive sufficient CO_2_ for photosynthesis. The area of chloroplasts that are lining the mesophyll cell membrane is strongly correlated with mesophyll CO_2_ conductance (Evans et al. 1994) and photosynthetic capacity (Oguchi et al. 2003, 2005).

In contrast to acclimation, among-species variations in LMA are associated with tissue density as well as leaf thickness. Poorter et al. (2009) suggested that the relative contribution of tissue density and thickness is 80:20 in explaining among-species variations of LMA. On the other hand, in the dataset of Onoda et al. (2011) containing 1698 species-site combinations, the relative contribution of tissue density and thickness was 46:54, meaning that the contribution of tissue density and thickness are comparable to one another (Y. Onoda, unpublished results) (Fig. 5c). These differences are associated with the biomechanical properties of leaves. Higher LMA indicates a greater force to shear (i.e., greater mechanical resistance of the leaf to braking forces), whether it is exerted by wind, abrasion, or herbivores (Wright et al. 2005a; Onoda et al. 2011). Regardless of the reasons, such as growing season or nutrient limitation, if a longer LL is favored, the leaves must be sufficiently tough with an investment in cell walls and structural strength to endure environmental stresses during the leaf lifespan. Shade-adapted evergreen species tend to have long-lived leaves (because otherwise, it is difficult to recuperate the cost of leaf construction) and high LMA compared with early successional and sun-adapted evergreen species (e.g., Kitajima 1994; Walter and Reich 1999).

A higher allocation to cell walls in a leaf has pros and cons. One advantage of greater cell wall content is greater toughness and mechanical support, which are best achieved with high cellulose and lignin concentrations. Among species with differing LL, high LMA leaves tend to have a higher proportion of leaf mass in cell walls in general, as well as thicker mesophyll cell walls (Onoda et al. 2017). Thicker mesophyll cell walls slow CO_2_ diffusion and limit the CO_2_ assimilation rate (Evans et al. 2009; Terashima et al. 2011; Flexas et al. 2012). Another disadvantage to photosynthesis in high LMA leaves is the proportion of N allocated to the cell walls (Onoda et al. 2004; Takashima et al. 2004; Hikosaka and Shigeno 2009). Although the plant cell wall consists primarily of non-nitrogenous materials, such as cellulose, hemicellulose, and lignin, it also contains specialized proteins, such as proline- and hydroxyproline-rich glycoproteins (Carpita and MaCann 2000). Consequently, nitrogen allocation to the photosynthetic machinery is proportionally lower in leaves with greater LMA, which is negative for photosynthetic productivity per se. On the other hand, mechanical support by allocation to cell wall in thicker leaves produces space to enable a greater mesophyll surface area as described earlier. These pros and cons relative to inter-specific variations in LMA appear to be largely in balance, as indicated by no clear trend of *A*_area_ among species against widely different LMA (Onoda et al. 2017; Fig. 2). Because of the lower CO_2_ diffusion and smaller investment in the photosynthetic machinery, high LMA species exhibit lower PNUE (Hikosaka et al. 1998; Poorter and Evans 1998; Onoda et al. 2017; Xue et al. 2023). Negative correlations were observed between PNUE and LMA as well as PNUE and LL across species (Hikosaka 2004; Fig. 2). This may be associated with a trade-off between NP and MRT as discussed above. Therefore, leaf trait variations are strongly associated with the whole-plant level NUE.

Similar conclusions were reached by Osnas et al. (2013), who treated these contrasting patterns of leaf traits relative to intra- and inter-specific variations with a statistical partitioning approach. They considered that the intra- and inter-specific variations of whole leaf photosynthesis are correlated better with total leaf area and leaf mass, respectively (i.e., area-proportional vs. mass-proportional). From the analysis of within- and among-species variations in two tropical forests, Osnas et al. (2018) found that the whole-leaf photosynthetic capacity is largely area-proportional when multiple species are compared, whereas it is largely mass-proportional for within-species variations. These statistical patterns depicted as area-proportionality vs. mass-proportionality may be explained by empirical data comparing LMA, leaf thickness, tissue density, and nitrogen relative to anatomical, physiological, and biomechanical traits of leaves among and within species.

## Temporal changes in photosynthesis and leaf traits

Both in temperate and tropical regions, various leaf production patterns are found among species (single flush, multiple flushes, sequential addition, and turnover). Many deciduous species develop their leaves early in the season favorable for growth. In temperate latitudes, leaves of deciduous plants begin to emerge in the spring and become mature over several weeks. In tropical latitudes, leaves of many deciduous trees emerge and develop early in the wet season, with time required for their expansion and physiological maturation differing widely among species from a few weeks to a few months.

In temperate region, photosynthetic rates in situ are relatively higher in the summer and lower in other seasons. This may be explained by two reasons. One is that the photosynthetic rates increase with temperature up to the optimal temperature (*T*_opt_), which is observed in both deciduous and evergreen species (Berry and Björkman 1980; Hikosaka et al. 2006; Yamori et al. 2014; Kumarathunge et al. 2019). In addition, the *T*_opt_ is generally higher at higher growth temperature (GT) (Yamori et al. 2014; Kumarathunge et al. 2019). Therefore, plants that adjust *T*_opt_ to achieve higher photosynthetic rates during the warmer season. Acclimation ability of photosynthesis to GT, however, may be limited in warmer regions. Comparing *Quercus serrata* plants growing near the northern and southern limits, Yamaguchi et al. (2019) report that *T*_opt_ would not change when the daily maximal temperature is higher than 30 °C. Hence, *Q. serrata* plants near the southern limit may not tolerate future warming. Similarly, Doughty et al. (2023) indicated that tropical forests are approaching critical temperature thresholds.

**Fig. 6:**
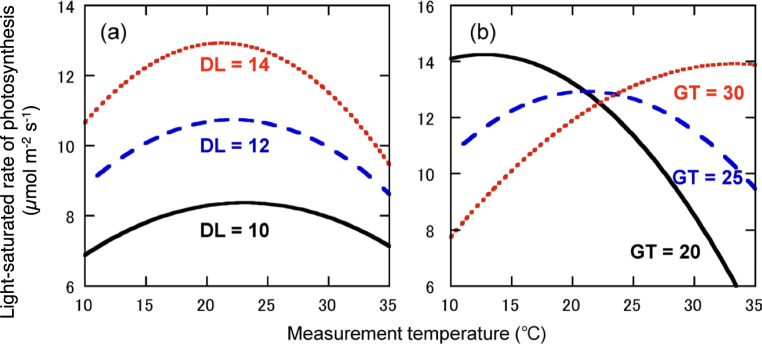
Simulated effects of daylength (DL, **a**) and growth temperature (GT, **b**) on the temperature response curve during photosynthesis. Growth temperature was fixed at 25 °C and the photosynthetic rate was calculated at various temperatures using three DLs (**a**). Day length was fixed at 14 h and A was calculated at various temperatures using three GTs (**b**). Photosynthetic rate was reconstructed based on multiple regression analysis of the maximum carboxylation rate of rubisco (*V*_cmax_) on DL and GT. When only DL is altered, the shape of the curve is similar but the intercept (height of the curve) changes, whereas GT is altered, absolute rate of photosynthetic rate at 23 °C was similar but the slope (temperature dependence) was different. Redrawn from Yamaguchi et al. (2016)

The other reason for the seasonal change in the photosynthetic rates is the dependence of photosynthetic capacity on leaf ontogeny in deciduous species. In general, *A*_area_ increases during leaf development, and, after the maturation, it decreases until fall (Kramer and Kozlowski 1979; Reich et al. 1991b). Previous studied have shown that the temporal decline in *A*_area_ is influenced mainly by day length (DL) (Busch et al. 2007; Bauerle et al. 2012; Stoy et al. 2014). Because DL and temperature are roughly correlated in natural environments, their effects may be confounded, but warming experiments can help to distinguish these effects. Yamaguchi et al. (2016) conducted a warming experiment with the canopy leaves of a deciduous tree (*Q. serrata*) using canopy open-top chambers installed in a cool temperate forest. The *T*_opt_ of photosynthesis was positively correlated with GT, whereas the photosynthetic rate at 20 °C was positively related to DL but not GT (Fig. 6). Thus, in this experiment, GT and DL affected different aspects the temperature dependence of photosynthesis, namely, the ‘slope’ and ‘intercept’ of the response curve (Fig. 6).

In the tropics, the optimality of seasonal changes in leaf photosynthetic traits may be explained mainly by water availability, but also by seasonal changes in cloud cover and light availability. Early in the dry season, when there is still sufficient moisture in the soil with more light from less cloud cover, there is a window of opportunity for photosynthetic production to canopy trees (Graham et al. 2003). Curiously, leaves that start to develop before the onset of the dry season and the significant increase in light results in significantly higher LMA and *A*_area_ compared with those that develop early in the rainy season (Kitajima et al. 1997b). These pre-dry season leaves have a much shorter LL in the direction that fits in the LES both in deciduous and evergreen species. The change of DL is very small, and seasonal decrease of cloud cover in the late wet season is gradual and unreliable. Hence, what environmental signals prompt this phenotypic plasticity response remains unknown.

In temperate or dry forests, evergreen leaves with LLs longer than one year experience seasonal environmental changes to which they exhibit acclimatory responses. At higher latitudes in the temperate zone, evergreen broadleaf shrubs in the forest understory maintain leaves closer to the ground to avoid freezing and freeze-induced embolism. These evergreen understory species exhibit large fluctuations of *N*_area_ and *A*_area_ within a year (Kume and Ino 1993; Skillman et al. 1996; Miyazawa and Kikuzawa 2005). In such species, it is reported that *A*_area_ at the GT is higher in summer compared with that in winter, whereas *N*_area_ tends to be higher during winter (Miyazawa and Kikuzawa 2005; Muller et al. 2005). Muller et al. (2005) examined seasonal changes in leaf traits in an understory shrub *Aucuba japonica* under three light regimes (gap, understory in a deciduous forest, and understory in an evergreen forest) and reported positive and negative correlations of *N*_area_ with light availability and GT, respectively. This may represent an adaptive strategy to take advantage of the greater light availability during winter in a deciduous forest. Indeed, the optimal *N*_area_ that should maximizes daily PNUE (**B** in Fig. 4) is predicted to be higher and lower at higher light availability and higher temperature, respectively, and observed *N*_area_ measured throughout one year corresponded to this prediction (Muller et al. 2011).

The optimization of *N*_area_ may also be related to the phenology of evergreen plants (Muller et al. 2011). A higher *N*_area_ is advantageous in winter when light availability and temperature are higher and lower, respectively. However, this advantage disappears in the summer when plants are shaded by a canopy of deciduous trees and the temperature is higher. The excess nitrogen may be reallocated to the construction of new leaves, fruit and seed. Newly absorbed N from the roots after summer is used to increase *N*_area_ for the winter. Compared with the understory plants, seasonal changes in the upper canopy leaves of evergreen trees are not necessarily obvious, because both light and temperature decrease in the winter. However, Han et al. (2008) observed that the *N*_mass_ of the canopy leaves of *Pinus densiflora* slightly increased toward winter, which may be explained by the allocation of newly absorbed N to be stored in existing evergreen leaves until shoot growth starts in the spring.

It may be generalizable that evergreen species do not seasonally accumulate nitrogen in their stems and roots (Millard and Grelet 2010), because evergreen leaves can serve as a nitrogen storage organ during the season not favorable for photosynthesis. Such nitrogen stored in evergreen leaves may be subsequently allocated to a new cohort of leaves at the seasonal flush. In contrast, deciduous trees use nitrogen stored in the stems and roots for seasonal flush of new leaves, which is likely supplemented by de novo uptake of nitrogen from the soil and decomposing leaf litter. This is a potentially important issue associated with resource competition between evergreen and deciduous species.

Leaf aging also has a strong influence on leaf traits. In herbaceous species, which have been more intensively studied than trees, *A*_area_ and *N*_area_ increase rapidly during leaf expansion, reaching their highest levels (i.e., physiological maturity) sometime after full leaf expansion, and declining thereafter linearly with time (Thomas and Stoddart 1980). With leaf age, *A*_area_ and *N*_area_ decrease simultaneously as the photosynthetic proteins become degraded, and nitrogenous compounds, such as amino acids, are translocated to newer developing leaves and other organs (Makino et al. 1983). Thus, it is widely accepted that leaf senescence is a process to recycle nutrients within a plant (Thomas and Stoddart 1980; Smart 1994). Conversely, in woody species, the leaf-age-dependent decline of *A*_area_ and *N*_area_ may be less generalizable. A parallel decline in *A*_area_ and *N*_area_ has been reported from some tree species (Field et al. 1983; Kitajima et al. 1997a, b), but there are also cases of decreasing* A*_area_ without significant changes in *N*_area_ in deciduous trees (Wilson et al. 2000, 2001; Hikosaka et al. 2007) and evergreen species (Escudero and Mediavilla 2003), which results in an age-dependent decrease in PNUE. Nitrogen resorption primarily occurs during the short period before the leaves are going to die. Niinemets et al. (2005) found that a decline in mesophyll conductance is involved in a decrease in *A*_area_ and PNUE. However, physiological and biochemical changes in senescing leaves of woody species and their inter-specific diversity remain less generalizable compared with those of herbaceous species.

## To shed, or not to shed? Optimal timing of leaf shedding

Leopold (1961) categorized leaf senescence into four types, ‘overall senescence’ in monocarpic species, ‘top senescence’ in perennial herbs, ‘deciduous senescence’ in deciduous woody species, and ‘progressive senescence’. In the top and deciduous senescence, plants lose all leaves during unfavorable season to avoid freezing or drought-induced wilting. Deciduous senescence is generally cued by specific environmental changes. Estiarte and Peñuelas (2015) reviewed potential environmental cues for deciduous senescence and suggested that photoperiod and temperature are the main environmental signals in winter-deciduous species, with water stress having an additional influence. Sensitivity to these cues varies among species. For example, at higher latitudes, there is a trend that the photoperiod is more important, whereas temperature is less important (Estiarte and Peñuelas 2015).

For progressive senescence, there are several theories or concepts to predict the optimal timing of leaf senescence, as reviewed in the earlier sections. If there are no limitations in resource availability and the number of leaves, then plants should retain each leaf until its carbon gain reaches zero (when *G* is maximum in Fig. 3a; Monsi and Saeki 1953; Saeki 1960; Chabot and Hicks 1982; Ackerly 1999; hereafter 'C-balance model'). The Kikuzawa model predicts that if there is a limitation in the total number of leaves, older leaves drop before carbon gain is higher than zero (when *g* is maximum in Fig. 3a; Kikuzawa 1991). If nitrogen availability limits plant growth, an old leaf may be dropped when the ratio of PNUE of the old leaf to that of the new leaf is lower compared with the resorption ratio (Escudero and Mediavilla 2003; Eq. 6; hereafter 'N-limitation model').

Which of these models explains actual leaf shedding? Ackerly (1999) examined leaf dynamics in the shoots of the tropical pioneer tree species. Actual LL in these species was longer than the optimal LL predicted by the Kikuzawa model, and these species retain their leaves until the leaf carbon balance reaches zero, which is consistent with the C-balance model. Oikawa et al. (2006, 2008) established experimental stands of an annual *Xanthium canadense* at two different nutrient levels. They found that the threshold of leaf shedding varied depending on nutrient availability. In the high nutrient stand, the leaves were retained until their daily carbon gain reached zero, which was consistent with the C-balance model. In contrast, in the low nutrient stand, leaves were shed while the carbon gain remained positive in a manner consistent with the N-limitation model by Escudero and Mediavilla (2003). Hence, plants may change the optimization rules to shed old leaves depending on the environment. When nitrogen availability is limited, leaf shedding occurs as the N-limitation model predicts, while when nitrogen is no longer a limiting factor, leaf is retained until carbon balance becomes zero (the C-balance model). Although Oikawa et al. (2006) did not calculate the optimal LL predicted by the Kikuzawa model, it can be estimated from the time dependence of cumulative carbon gain for each leaf, which is shown in Fig. 6 of Oikawa et al. (2006). The optimal LL was 1–2 weeks for most leaves, which was much shorter than the actual LL. This suggests that the Kikuzawa model is not applicable to these stands.

In the C-balance model, the optimal timing for leaf shedding depends on the traits and environment of the shedding leaf only, whereas in the N-limitation model, the optimal timing depends not only on the PNUE of shedding leaf but also on PNUE of young leaves (see Eq. 6). Therefore, in the latter model, low PNUE of the focal leaf is not a requirement for shedding; if PNUE of young leaves is also low, the focal leaf should be retained. Therefore, which of C-balance or N-limitation model is effective can be judged by comparison of partial and whole shading treatments. If leaf shedding is determined by C-balance, a leaf would be shed both when all leaves of the individual are shaded (whole shading) and when part of leaves are shaded but others are unshaded (partial shading). In contrast, if leaf shedding is determined by N-limitation, shaded leaf in the partial shading would be shed because its PNUE is lower than that in unshaded leaves, but shaded leaves in the whole shading would be retained because all leaves have similarly low PNUE. Yoshimura (2010) demonstrated that whole shading elongated LL of old leaves compared with partial shading in the branches of *Cedrela sinensis* growing in the natural environment. In addition, Hikosaka et al. (2010) also experimentally demonstrated that LL of an old leaf is explained by relative difference in light environment between young and old leaves, rather than the absolute light environment of old leaves, in an evergreen understory herb, *Viola hondoensis*. These results also suggest that the N-limitation model is effective in natural environment at least in some plants.

The above-mentioned studies suggest that the Kikuzawa model, which assumes that a leaf is shed when the marginal gain *g* is maximized, is not strictly applicable to the real plants. As mentioned above, this model assumes that the leaf number of a plant (or shoot) is constant. This situation may not be applicable to experiments in which the studied plants are often small for convenience of the measurements and manipulations. Plants likely shed their old leaves based on the C-balance or N-limitation model when plants are small, whereas the Kikuzawa model may be applicable when they become mature. This may explain why the Kikuzawa model well explains global patterns of leaf habits and traits. However, experimental evidence is still limited to generalize this idea.

Xu et al. (2017) conducted a simulation to show that the improved Kikuzawa model can quantitatively predict LL of tropical species. A decline in photosynthetic rate (*b* in Eq. 3) was strongly correlated with *V*_cmax_ (maximum carboxylation rate) per unit leaf mass across various species. They incorporated the *b*-*V*_cmax_ relationship, LMA, biochemical model of photosynthesis, and light environment of 105 Panamanian species into the Kikuzawa model. The actual LL was strongly correlated with the optimal LL, which explained 40% of the variation in the actual LL.

## Return on investment: can leaf carbon gain explain the coexistence and replacement of species?

Since plant fitness strongly relies on photosynthetic carbon gain, we expect that species with leaves that gain more carbon are advantageous through natural selection. At a leaf level, higher lifetime return on investment (RI), i.e., the amount of carbon assimilated through lifetime per unit invested carbon for construction of the leaf, may represent a proxy of fitness. Species with lower RI is thus expected to be eliminated from the environment through competition with other species. In other words, coexisting species are expected to have similar RI to each other.

RI should be higher if the leaf has higher instantaneous photosynthetic rate, smaller investment, and/or longer LL. Since construction cost (energetic costs for tissue construction per leaf mass) is not very different among species (Chapin 1989; Wiliams et al. 1989; Villar and Merino 2001), *A*_mass_ can be regarded as a measure of the ratio of the instantaneous photosynthetic rate to the investment. Then, a question arises that which species with higher *A*_mass_ or those with longer LL have higher RI along leaf economics spectrum. Westoby et al. (2000) indicated that the slope of the log–log relationship between LL and *A*_mass_ was smaller than − 1. This indicates that LL multiplied by *A*_mass_ is greater in species with a longer LL, leading to a next question why there are many species with different LLs can coexist.

Westoby et al. (2000) considered that equal-benefit is necessary for coexistence and the lower *A*_mass_ × LL in short LL species is compensated by the time-discounting effect. This concept assumes that carbon acquired earlier is more valuable than that acquired later. They raised four factors associated with the time-discounting effect: (1) the reduction of photosynthetic rate because of overshading by surrounding vegetation and self-shading, (2) reduction of photosynthesis because of the accumulation of algae, fungi, and damage from the herbivory, (3) the risk that the plant dies before leaf death, and (4) the compound-interest effect. The compound-interest effect means that earlier photosynthates can be reinvested to generate new leaves, resulting in greater carbon gain at the plant level compared with the simple interest from a leaf. This concept was first indicated by Blackman (1919) at the plant growth level, and then, by Harper (1989) at the leaf level ("the value of a leaf"). Subsequently, Falster et al. (2012) revisited this idea and proposed three hypotheses to explain why shorter LL species can coexist with longer LL species: (1) the decline of the photosynthetic rate due to leaf aging or mutual shading by younger leaves, (2) covariation among traits to offset the disadvantage of a shorter LL, and (3) the compound-interest effect. They separated the first two hypotheses from the third one, designating the latter as the time-discounting effect.

Many other studies also considered leaf lifetime carbon gain empirically to see whether RI is similar across species to explain the coexistence of multiple species, and/or whether the time-discounting effect needs to be incorporated. Mediavilla and Escudero (2003) determined LL and age-dependence of *A*_mass_ in deciduous and evergreen Mediterranean species. LL × *A*_mass_ was greater in longer LL species when *A*_mass_ of the youngest leaves was used for the calculation, as done by Westoby et al. (2000). However, when *A*_mass_ was averaged over the lifetime of the leaf, LL × *A*_mass_ was independent of LL. Kikuzawa and Lechowicz (2006) compared data for 26 species compiled from the literature obtained in a variety of sites, biomes, and climate zones to calculate RI. They found that there was no relationship between RI and LL. Selaya and Anten (2010) compared the leaf lifetime carbon gain among early (shorter LL) and late successional species (longer LL) in a tropical forest secondary succession and found no relationship between RI and LL. This was because a decline in light availability with leaf age was greater in the late successional species. Using five pairs of coexisting deciduous and evergreen woody species, Ye et al. (2022) studied deciduous and evergreen plants in temperate climate and calculated their leaf lifetime carbon gains through monthly measurements of the photosynthetic rates, which were compared against their leaf construction costs. Evergreen species achieved a higher leaf lifetime carbon gain but also had higher leaf construction costs compared with deciduous species, which resulted in a similar RI between deciduous and evergreen species. Thus, these four studies suggested that the coexistence of various strategies may be explained without the compound-interest effect. Conversely, Falster et al. (2012) examined saplings of 10 Australian woodland species and found that a greater RI was associated with a longer LL species. In the simulation study of Xu et al. (2017) using a tropical rain forest dataset (see also the previous section), the predicted RI was also greater in longer LL species. The latter two results suggest that the coexistence of species with different LLs cannot be explained without the compound-interest effect.

How can we explain these discrepancies among these studies? Here we propose two factors: the first is that the compound-interest effect may be applicable only when the leaf number of a plant does not reach its maximum. Earlier carbon gain is advantageous because it contributes to the production of new leaves and the plant can have a higher growth rate because of a large number of leaves (Harper 1989). Conversely, if the leaf number reaches a maximum, the production of new leaves is accompanied by the shedding of old leaves. The value of earlier carbon gain is not higher compared with later carbon gain. Therefore, the effectiveness of the compound-interest effect differs depending on whether leaf area development reaches a maximum or not. The second factor is the disturbance effect, which is important for species coexistence in forest ecosystems (Hubbel 2001). In forest understory, RI is suppressed due to shading by canopy trees. As suggested by the intermediate disturbance hypothesis of Connell (1978), pioneer and early successional species can persist in a forest when high resource patches are created by disturbance. Shorter LL species include shade-intolerant species and early successional species (Reich et al. 1991a). Because treefall gaps are formed at certain frequency in most forests, species with shorter LLs may be able to coexist with longer LL species despite their lower RI.

**Fig. 7:**
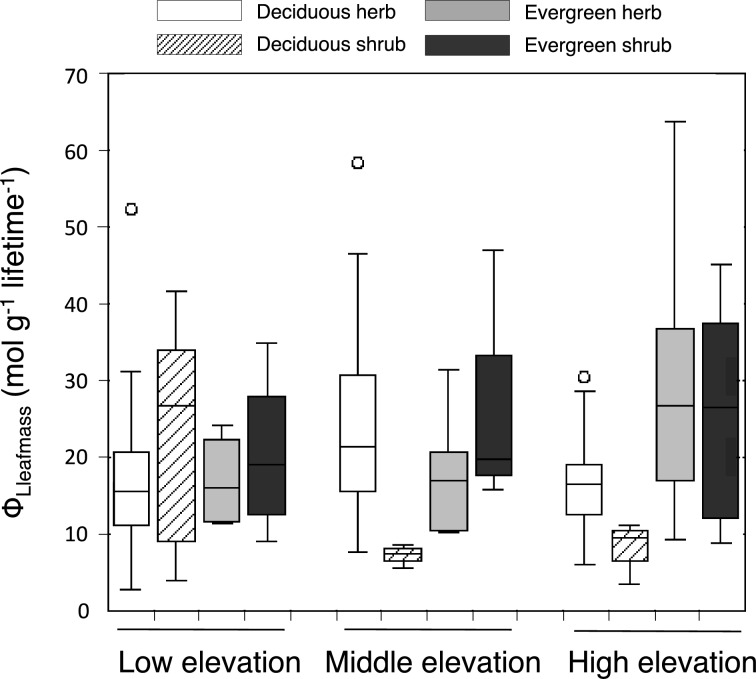
Lifetime light absorption per unit leaf mass (Φ_Lleafmass_) of different functional groups in the moorlands at different altitudes. Open, hatched, gray, and black columns denote deciduous herbs, deciduous shrubs, evergreen herbs, and evergreen shrubs, respectively. The four leftmost columns denote low altitude (600 m), the four rightmost columns denote high altitude (1300 m), and the middle four columns represent middle altitude sites (1000 m). The horizontal lines on the columns denote the 25th, 50th, and 75th percentile values, and the whiskers represent the range of values. Redrawn from Kamiyama et al. (2014) with modifications

Concerning the disturbance effect, the alpine or high-latitude moorland is an interesting ecosystem because multispecies, including the deciduous and evergreen species, coexist for a very long time (often longer than 100 years) without obvious disturbances (Hikosaka et al. 2016b). Furthermore, species composition changes along environmental gradients, such as temperature and pH, among moorlands (Sasaki et al. 2013). Therefore, we can determine whether RI is required to be equal for coexistence in a given environment. Kamiyama et al. (2010, 2014) investigated light absorption, biomass allocation, and LL of plant species coexisting in moorland plant communities at various altitudes. In mid-summer, when the stand biomass was maximal, light absorption per leaf mass was smaller in evergreen than in deciduous species because they are shaded by taller deciduous species (Kamiyama et al. 2010). However, the evergreen species receive strong light in the spring because the leaves of the deciduous plants are not present. This results in seasonal partitioning of light interception between the deciduous and evergreen species. A longer LL in evergreen species also contributes to lifetime-light interception. As a result, the leaf-lifetime light-acquisition efficiency (Φ_Lleafmass_; lifetime-light acquisition per unit leaf mass) was not different between deciduous and evergreen species at low altitudes (Fig. 7). Furthermore, Φ_Lleafmass_ could explain replacement of species along altitude; at a higher altitude, the evergreen species had a higher Φ_Lleafmass_ than the deciduous species (Fig. 7) due to a smaller shading by dominant deciduous species. The lifetime carbon gain per unit leaf mass (close to RI) was not significantly different between deciduous and evergreen species at the high altitude, whereas it was slightly lower in evergreen species at low altitude (C. Kamiyama, unpublished data). This result is consistent with the fact that the number of evergreen species was smaller at lower altitudes in the studied area (Hikosaka et al. 2016b).

Considering the above results, we propose a hypothesis on the relationship between RI and the coexistence of species with different LLs. If there is no environmental suppression, longer LL species are advantageous because they have higher RI values compared with shorter LL species. In nature, however, suppression of RI occurs due to environmental stressors and/or species interaction such as shading by canopy plants. Shorter LL species may be advantageous only when they grow in better environment than longer LL species (e.g., gaps in forests). They can rapidly occupy such open patches because of higher growth rates and the compound-interest effect. Over time, they will be eliminated from that patch via environmental changes or competition with longer LL species (e.g., gap closure and microscale secondary succession). In forests, depending on the disturbance regimes, they can persist in the community, at greater abundance when gap creation is more frequent. In the seasonal environment, shorter LL species may have equal or higher RI compared with longer LL species because the carbon gain of longer LL species is suppressed by unfavorable environment or species interactions. These situations make the deciduous species with shorter LL to be similar in RI to allow their coexistence with evergreen species with longer LL species.

This idea is consistent with a recent report that resource acquisitive species have lower growth rates than resource conservative species under most environmental conditions in nature, though they have higher *A*_mass_ (Augusto et al. 2025). They have higher growth rates only when environmental conditions are very favorable, but not under resource-limited conditions, due partly to shorter LL and lower RI. On the other hand, resource conservative species can maintain relatively high growth rates across a variety of resource availability, which might be achieved partly by a longer LL and thus longer mean residence time of absorbed nutrients.

## Conclusion

In a broad generalization, studies over the last 50 years have shown that deciduous leaf habit is a strategy to avoid seasonal risks of premature leaf loss from freezing or drought-induced embolism, whereas evergreen habit is to tolerate such stress to maximize carbon gain of the whole plant under resource limitations. Geographically, their relative abundance changes with climate (duration of unfavorable periods for photosynthesis) and soils (evergreen species favored in infertile soils). Within a site, disturbance regimes in a plant community can promote their coexistence, as gaps in vegetation makes it possible for deciduous species with short-living leaves to achieve similar return on invest as evergreen species. These patterns at different spatiotemporal scales can be explained from the perspective of optimization of leaf traits, which exhibit continuous variation along the leaf economics spectrum. Relatively underappreciated in the literature is the function of evergreen leaves as the site of seasonal nutrient storage, which would require simultaneous assessment of age- and season-dependent changes of leaf nutrient contents. Quantitative models of leaf lifespan continue to be useful guidance principle to predict how plant communities respond to changing climate or changes in disturbance regimes. However, the optimal solution differs depending on the factor that limits the amount of the leaves in the canopy. The limiting factor may differ between species, and between environments. Which model is applicable to the respective vegetation is still unclear and further studies are necessary. Empirical studies to identify cases that do not fit perfectly to these patterns help uncover yet-to-be recognized adaptive traits relevant for variations in leaf lifespan and leaf habit.

## Data Availability

Not applicable.
